# Methods and Manipulations with Gold in Filling Teeth

**Published:** 1882-08

**Authors:** W. F. Morrill

**Affiliations:** New Albany, Ind.


### METHODS AND MANIPULATIONS WITH GOLD IN FILLING TEETH.

BY W. F. MORRILL, NEW ALBANY, IND.

[Read before the Indiana State Dental Society.]

It is not my intention to traverse the entire history of methods and manipulations with gold in filling teeth. I propose to quote some of the processes adopted by our representative men from the earliest days down to the present, and to draw such deductions as shall enable us to observe what advancement in this particular department has been made in our profession.

Method implies system, order, arrangement or process, by which an object is accomplished. In the operation of filling a tooth, a clear and well defined method of procedure is not only necessary, but the human hand and eye, educated and trained, are important factors of success. A clear vision and a responsive hand are of essential value. By these the mysteries of a carious tooth are unfolded and the precise condition of the tissues revealed and conveyed to the judgment of the operator. If you would learn how keen the eye and how responsive the hand become under severe training, go to the feet of an Allport, Atkinson, Hunt, Taft, Webb, McKellops, or any other high priest of our profession and there learn your humiliation. The difference between these masters and the ordinary operator is as the bow of Ole Bull in one of his exquisite masterpieces of execution and that of a common street-fiddler. All practitioners cannot have the celerity, grace and inspiration of these gentlemen already named, but by hard endeavors and due diligence much may be accomplished toward these attainments.

Gold for filling teeth has stood the test of experience longer than any other material, notwithstanding "New Departure," "New Creeds" and old cranks. Its virtues are manifold. Its imperishable lustre is undimmed by the cycles of ages. It enriches the arts. It is the emblem of royalty and power. It exalts the dignity of manhood by giving it tone and respectability. No dentist ever felt degraded by using gold in his operations.

Think of it, how long and close are associations with gold. What a constancy and faith have grown up between us—an attachment and confidence from which we are not easily shaken nor severed.

It requires, commercially speaking, some nerve to handle gold; and yet when placed in a tooth in proximity to nerve none have fortitude enough to bear it!

For saving teeth gold foil has a fair record—better than anything else. Other forms of gold, such as crystal, Lamm's Morgans, cylinder, ropes, and other varieties and materials, have had one and all their praises sounded and their merits tested. And for the average operator the result of this experience teaches him to rely chiefly on gold foil. It is true various preparations of gold have had their working qualities much improved and none of them are without advantages in filling teeth. We would not do without them. And yet the peculiar softness, purity and glove like texture of gold foil, must, among a majority of practitioners, forever remain their sheet anchor of dependence. It is true the manufacturers of gold foil better understand to-day the needs and properties required by dentists and have handsomely responded to these wants than in years gone by.

Forty years ago, Dr. Paul B. Goddard, of University of Pennsylvania, published an illustrated work on dentistry, which included "a treatise on diseases of the ivory," and comprising two or three paragraphs devoted to the method of filling teeth. I quote what he says in reference to the preparation of gold foil: " Aside from mastic and wax and fusible metal, there are tin and gold foil. The gold is prepared by cutting a strip an inch wide, then slit along the edge about every quarter inch ; this should then be wound into a rope. The cavity being dry and clean, one end of the rope is placed at the bottom of the cavity and with a small instrument it is folded over and over and round and round until the cavity is full. The pressure must be gentle and regular. It must bring the several layers in contact but never be so great as to cut through one layer or separate the little cord of foil. When the cavity is full it is formed into a heap, and with a blunt instrument condensed well into the cavity. It is occasionally useful to increase this pressure by a very small instrument which makes the metal very solid and causes it to hold well. After the excess of the metal is filed away, the filling is burnished with several burnishers, when the operation is complete.'

In 1848 Dr. J. D. White, of Philadelphia, under the fostering provisions of the publishers, Messrs. Jones, White and McCurdy, began a series of articles in the *Dental News Letter*, of which he was the editor. His were more intelligible methods, as well as more ingenious, than any which had previously been given. He bore at that time a fine reputation as an operator, and continued to conduct the *News Letter* with rare ability and zeal. In reference to manipulating gold foil, he says: "Cut No. 4 gold into one-fourth or one-half sheets, roll or fold; then twist them into a kind of rope but not crimped so as to break the leaf in either way; on the contrary, it should present as smooth a surface as possible. Some suppose the more roughly the rope is twisted the better, one fold when put into the cavity will hold upon another. But not so, besides, it makes a porous plug, and when the leaf is much crimped and broken, it cannot be rendered solid without much pressure; nor will receive so fine a polish." For facial cavities that are large, he would fold No. 4 foil over a watch spring about one-eighth inch wide in form of tape, so that when folded upon itself a kind of block is formed, then place in the bottom of cavity and finished with a rope of foil. This kind of block, nicely folded, is invaluable and indispensable in building up broken down sides of cavities, or placing along the cervical wall of large cavities where dampness is likely to permeate.

It should be our constant study to put the gold in such a condition before introducing it into the cavity as to be rendered solid with the least possible pressure and in the shortest possible time." He admonishes care in keeping the gold and cavity dry throughout the operation. It will herein be observed that Dr. White describes with more particularity his method of manipulating foil, which was in advance of many of his co-laborers.

During the next decade, when the DENTAL REGISTER, the *American Journal of Dental Science*, and perhaps a few other journals of dental literature, had found an existence, such apostles as Allport, Arthur, Badger, Blakesley, Cushman, Clark, Dwinelle, Harris, Goddard, of Louisville, Richardson, Taylor, Taft, Watt, Wescott, and a host of others, began to illuminate the dental world by ingenious methods, superior skill, and indomitable energy; then it was that shafts of intellectual light were thrown across our pathway which created those immortal principles of dental practice such as are the basis of our teachings to-day.

In 1855, Dr. Dwinelle published his method of manipulating crystal gold. It was the sensation of the day. It was heralded as a great triumph over gold foil; especially in restoring natural formations to teeth broken away. It was the beginning of the contour filling era. Experience shortly after showed the material was uncertain in its properties, and that to make successful operations with it, required more skill and time than with gold foil. In short, crystal gold received a black eye of disappointment from which it never wholly recovered. The operators who now confine themselves to this variety of gold are few. By habits of long experience with it they no doubt do acceptable work.

As the period which I am mentioning, the cylinder fillings of Dr. Jno. S. Clark and Dr. Jas. Taylor, were attracting attention throughout the South and West. Dr. Clark was generous to communicate to his professional brethren his method of forming the cylinders and of introducing them into the cavity. Dr. Taylor, as the editor of the DENTAL REGISTER, also gave his manner of filling teeth with cylinders. Dr. Clark was on friendly intercourse with Dr. Badger, of Nashville, a most distinguished dentist of that day, and communicated his cylinder method to him; and yet Dr. B., although practicing a similar method, did not return the compliment of giving his manipulation processes with gold. He, however, condescended to publish his method, which was the only article I find of his in dental journals. This was in 1856, he says, "Take Abbey's gold foil No. 4, fold it into a strip one-third wider than the cavity was deep. The strip is then folded end wise upon itself until it could be folded no longer, then roll in the hand or finger. The proper amount of foil having been used, a pellet was formed the thickness of a bur drill which had been used, its length being the same as the width of the strip out of which it had been formed. It was then forced through a smooth hole in a steel plate a little too small to allow the bur to pass through, in order to ensure its ready entrance into the cavity it was to fill. Then anneal and place conveniently by for use." The word "anneal" applied to gold foil, I do not, previous to this, find elsewhere, as a practice. The disclosure of these methods to the toiling student of dental practice was a bright epoch, for college advantages were few, and the sources of information to qualify oneself were limited.

Between 1850 and 1860 were years of great dental progress.

Novelties in new instruments and ingenious methods multiplied considerably. The special qualities of gold began to be better understood and the instruments for manipulating foil were better adapted for its use.

About this time Dr. Wescott had found "sticky" or cohesive foil. And so had Dr. Leslie almost simultaneously with Dr. Wescott. We have the statement of both of these gentlemen in regard to the priority of the discovery, and it is perhaps due to their memory, as both are deceased, to say that their good names ought not to be tarnished by calling attention to this virtue in gold. Along with the discovery of cohesive gold Dr. Robt. Arthur, the most accurate and painstaking writer of that period, introduced serrated pluggers for filling teeth, and this acquisition was followed later by Dr. Atkinson's mallet which has since been partially superceded by the electric mallet, esteemed the greatest masterpiece of dental triumphs. These were the beginning of the high art period of American dentistry.

It cannot be claimed to-day that contour fillings, or decorative dentistry, so called, is growing in public favor, or is as popular as it was. Mallets are not swung with the vigor they once were; cohesive gold is not universally preferred over soft foil; nor are pluggers bearing much beyond smooth surfaces or only slight serrations, growing popular. To keep one's self abreast and alive in all things originating for the more perfect methods of manipulating gold, is a commendable ambition that should be encouraged. Yet at the risk of abandoning methods that have proved available in one's hands and not incur mortification over bad operations, changes should be carefully considered before attempting them. Rather than failures to happen, better suffer the stigma of old fogy and have your methods crowned with success. For the past few years in methods of manipulating gold we have had, in original novelties, comparative rest. This is a good sign. It is an indication of better work being done and more teeth saved. The properties of gold foil are now better understood and the treatment it receives at the hands of both maker and operator, is nicer and more intelligible. Gold despises " unmannerly" thrusts, but it does not object to have its talents rolled in a napkin.

It is not infrequent to meet with dental practitioners who have superior methods of manipulating foil. If there are any who have not, their modesty has not yet been reported.

The numerous expert operators and the able journals at our command, afford ample opportunities to know the present status of manipulating gold ; yet, at the risk seeming familiar. I shall recall a few of the latest modes, advocated by some of our best men. Within a very recent date Dr. Shumway offers some novelties in cylinders with points, but in fact resembling pellets. He takes a sheet of gold foil, cuts into four squares, folds one-quarter or one-half inch, or less if you wish ; fold the corners together, making a triangle, place the broach in the center of the triangle and roll it into a solid cone. This form he uses in the first part of the filling as any dentist would. After a certain portion of the cavity is filled in this way, he cuts a leaf into small squares of single thickness of No. 4 gold with ivory points, he burnishes the filling already begun. It is claimed by this method more perfect adaptation of gold to the walls of the cavity. Dr. La Rouche prefers to have his cylinders made by rolling No. 2 or 3 soft foil on a piece of steel long enough to roll a whole sheet ; then with No. 4 or 5 cohesive gold foil is wound outside of this cylinder which holds it together nicely. After withdrawing the steel he cuts them into desired lengths with a razor. The cohesive covering on the outside is said to be an improvement, leaving the inside soft which is a beautiful condition to work.

Dr. A. A. Blount, of Geneva, whose reputation seems to have not grown dim by the effete despotisms of Europe, bespangles us with the merits of a new method that is said to bring forth first-class results. He details his manner of operating, using both soft and cohesive foil in the same cavity. As soft is better adapted to the walls of the cavity, he begins with that, and in a line of the periphery, filling the center with cohesive gold. All the manipulations are done with smooth-pointed pluggers, made by himself.

In the beginning of a dental practice if one has been taught a method of using gold serviceably and longer experience confirms his experience, let him not forsake it without something better and due deliberation. In conclusion let me emphasize one single fact in manipulating foil, " Be sure in impacting gold around the circumference of the cavity, you have made it so that no lodgement of moisture shall find entrance." Do this and you will have "no more worlds to conquer. You will then prosper and be happy. You will be among the elect."

